# Feeling How Old I Am: Subjective Age Is Associated With Estimated Brain Age

**DOI:** 10.3389/fnagi.2018.00168

**Published:** 2018-06-07

**Authors:** Seyul Kwak, Hairin Kim, Jeanyung Chey, Yoosik Youm

**Affiliations:** ^1^Department of Psychology, Seoul National University, Seoul, South Korea; ^2^Department of Sociology, Yonsei University, Seoul, South Korea

**Keywords:** subjective age, self-perceptions of aging, gray matter atrophy, VBM, brain age

## Abstract

While the aging process is a universal phenomenon, people perceive and experience one’s aging considerably differently. Subjective age (SA), referring to how individuals experience themselves as younger or older than their actual age, has been highlighted as an important predictor of late-life health outcomes. However, it is unclear whether and how SA is associated with the neurobiological process of aging. In this study, 68 healthy older adults underwent a SA survey and magnetic resonance imaging (MRI) scans. T1-weighted brain images of open-access datasets were utilized to construct a model for age prediction. We utilized both voxel-based morphometry (VBM) and age-prediction modeling techniques to explore whether the three groups of SA (i.e., feels younger, same, or older than actual age) differed in their regional gray matter (GM) volumes, and predicted brain age. The results showed that elderly individuals who perceived themselves as younger than their real age showed not only larger GM volume in the inferior frontal gyrus and the superior temporal gyrus, but also younger predicted brain age. Our findings suggest that subjective experience of aging is closely related to the process of brain aging and underscores the neurobiological mechanisms of SA as an important marker of late-life neurocognitive health.

## Introduction

Subjective age (SA) refers to how individuals experience themselves as younger or older than their chronological age. Subjective perception of aging does not coincide with the chronological age and shows large variability among individuals (Rubin and Berntsen, 2006). The concept of SA has been highlighted in aging research as an important construct because of its relevance to late-life health outcomes. Previous studies have suggested that SA is associated with various outcomes, including physical health (Barrett, 2003; Stephan et al., 2012; Westerhof et al., 2014), self-rated health (Westerhof and Barrett, 2005), life satisfaction (Barak and Rahtz, 1999; Westerhof and Barrett, 2005), depressive symptoms (Keyes and Westerhof, 2012), cognitive decline (Stephan et al., 2014), dementia (Stephan et al., 2016a), hospitalization (Stephan et al., 2016b) and frailty (Stephan et al., 2015a). Although chronological age is a primary factor in explaining these late-life health outcomes, these studies suggest that SA can be another construct that characterizes individual differences in the aging process.

The interoceptive hypothesis posits that a significant number of functions, both physical and cognitive, decline with age and this is subsequently followed by an awareness of such age-related changes (Diehl and Wahl, 2010). In other words, feeling subjectively older may be a sensitive marker or indicator reflecting age-related biological changes. This hypothesis is supported by several studies that have reported significant associations between older SA and poorer biological markers, including C-reactive protein (Stephan et al., 2015c), diabetes (Demakakos et al., 2007), and body mass index (Stephan et al., 2014). Moreover, the indices of biological age (MacDonald et al., 2011) including peak expiratory flow and grip strength, also were associated with SA, even after demographic factors, self-rated health and depressive symptoms were controlled for Stephan et al. (2015b). Among a variety of biological aging markers, a decrease in neural resource constitutes a major dimension of age-related changes in addition to physical, socio-emotional and lifestyle changes (Diehl and Wahl, 2010). Together with the interoceptive hypothesis, the subjective experience of aging may partly result from one’s subjective awareness of age-related cognitive decline. For example, subjective reports of one’s own cognitive decline have received attention as an important source of information for the prediction of subtle neurophysiological changes. Even when no signs of decline are found in cognitive test scores, subjective complaints of cognitive impairment may reflect early stages of dementia or pathological changes in the brain (de Groot et al., 2001; Reid and MacLullich, 2006; Stewart et al., 2008; Yasuno et al., 2015). It is, thus, possible to examine a link between the subjective experience of aging and neurophysiological aging.

To assess age-related brain structural changes and widespread loss of brain tissue, neuroanatomical morphometry methods have been widely used (Good et al., 2001; Fjell et al., 2009a; Raz et al., 2010; Matsuda, 2013). Moreover, the large neuroimaging datasets and newly developed machine learning techniques have made it possible to estimate individualized brain markers (Gabrieli et al., 2015; Cole and Franke, 2017; Woo et al., 2017). This approach can be advantageous for interpreting an individualized index for brain age, since predictive modeling can represent multivariate patterns expressed across whole brain regions, unlike massive and iterative univariate testing. In recent studies, estimated brain age was found to predict indicators of neurobiological aging, including cognitive impairment (Franke et al., 2010; Franke and Gaser, 2012; Löwe et al., 2016; Liem et al., 2017), obesity (Ronan et al., 2016) and diabetes (Franke et al., 2013).

Although SA has predictive values for future cognitive decline or dementia onset, few studies have examined the neurobiological basis of such outcomes. Combining both regional morphometry and the brain age estimation method, this study will provide an integrated picture of how each individual undergoes a heterogeneous brain aging process and supply further evidence of the neural underpinnings of SA (Kotter-Grühn et al., 2015). Using analyses for voxel-based morphometry (VBM) and age-predicting modeling, we aimed to identify whether younger SA is associated with larger regional brain volumes and lower estimated brain age. We also examined possible mediators, including self-rated health, depressive symptoms, cognitive functions and personality traits that were candidates for explaining the hypothesized relationship between SA and brain structures.

## Materials and Methods

### Subjects

The participants in this study were subsampled from the 3rd wave Korean Social Life, Health and Aging Project (KSHAP) which consisted of 591 older adults. KSHAP is a community-based cohort study that collected data from the entire population of older adults in Township K. In all, 195 elderly individuals received thorough health survey, psychosocial surveys and neuropsychological assessment. The health survey was conducted in 2014, and both the psychosocial survey and neuropsychological assessment were conducted in 2015. The following exclusion criteria were applied: psychiatric or neurological disorders, vision or hearing problems, having metal in the body that cannot be removed, hypertension and/or diabetes that cannot be controlled with drugs and/or insulin, and a history of losing consciousness due to head trauma. Older adults with cognitive impairment were excluded using neuropsychological tests and semi-structured interviews. The details of the screening procedures are described in a previous publication (Joo et al., 2017). The final data were comprised of 68 subjects who did not meet any of the exclusion criteria (mean age = 71.38, SD = 6.41, range = 59–84). MRI acquisition was done in 2015, and subsequent analysis was blinded from identification of all participants. The study was approved by the Institutional Review Board of Yonsei University, and all participants provided written informed consent to the research procedures.

### Subjective Age Groups

The following question was verbally asked to assess comparative SA: “How old do you feel, compared to your real age?” (Westerhof and Barrett, 2005). Participants responded with one of three categorized age identity options: “I’m younger than my real age” (younger SA), “I’m the same as my real age” (same SA) and “I’m older than my real age” (older SA; Boehmer, 2007). Among the subjects of KSHAP who did not have cognitive impairment (*n* = 137), those who identified themselves as younger SA were the greatest in proportion (40.1%), followed by same SA (34.3%) and older SA (25.5%). The gender ratio did not significantly differ among the three SA groups (*χ*^2^ = 4.324, *p* = 0.112).

### Cognitive Functions

The Mini-Mental State Examination for Dementia Screening (MMSE-DS; Han et al., 2010), category fluency test (Kang et al., 2012) and episodic memory and working memory indices from the Elderly Memory Disorder Scale (Chey, 2007) were used to assess cognitive functions. Category fluency test asked participants to generate words from the two semantic categories (i.e., supermarket and animal) each within a minute (Kang et al., 2000). The episodic memory index was calculated by adding the correct rates on the Long-Delay Free Recall of the Elderly Verbal Learning Test (EVLT), Delayed Recall on the Story Recall Test (SRT) and Delayed Reproduction on the Simple Rey Figure Test (SRFT). The EVLT is a nine-word learning test utilizing the California Verbal Learning Test paradigm (Chey et al., 2006). The SRT requires subjects to recall a paragraph containing 24 semantic units (An and Chey, 2004). The SRFT is a simplified version of the Rey-Osterreith Complex Figure Test modified for the elderly population (Park et al., 2011). All delayed recall subtests were administered 15−30 min after the immediate recall session. The working memory index was the sum of the longest correct backward digit sequence repetition span and longest correct Corsi-block tapping order (Song and Chey, 2006).

### Health and Psychosocial Covariates

We assessed potential mediators and covariates that could account for or confound the association between SA and brain structural characteristics. The covariates were selected on the basis of previously reported associations with self-rated health (Stephan et al., 2015b), personality traits (Stephan et al., 2012), cognitive functions (Stephan et al., 2014) and depressive symptoms (Keyes and Westerhof, 2012). Participants rated the level of their global health on a 5-point Likert scale: poor, slightly poor, good, very good and excellent. Higher values represented better self-rated health. Depressive symptoms were measured using the 30-items Geriatric Depression Scale (Yesavage et al., 1982), to which respondents indicated whether they had experienced a given symptom during the past week using a “yes” or “no”, i.e., on a binary scale. The personality traits of extraversion and openness were assessed using the NEO-Five-Factors-Inventory (Costa and McCrae, 1992) on a 4-point Likert scale, ranging from 1 (strongly disagree) to 4 (strongly agree).

### Voxel-Based Morphometry Analysis

Magnetic resonance imaging (MRI) scans were acquired in a 3Tesla MAGNETOM Trio 32 channel coil at Seoul National University Brain Imaging Center. Whole-brain T1-weighted magnetic-prepared rapid-gradient echo (MPRAGE) sequence images were acquired for each subject, with the following parameters: TR = 2300 ms, TE = 2.3 ms, FOV = 256 × 256 mm^2^, and FA = 9°. Whole-brain VBM analysis was carried out to determine the association between regional gray matter (GM) density and SA groups. The preprocessing of imaging data was conducted using the Statistical Parametric Mapping software (SPM12; Welcome Department of Imaging Neuroscience, London, UK) implemented in Matlab Version r2015b (MathWorks). T1 images were bias-corrected and segmented into five tissue classes, based on a non-linearly registered tissue probability map (Ashburner and Friston, 2005). The segmented native images were summed to infer individual total intracranial volume (TIV). To spatially normalize the GM image into the standard space with an enhanced accuracy of inter-subject registration (Ashburner, 2007), we used diffeomorphic anatomical registration using exponentiated lie algebra (DARTEL). A customized template was created, and a deformation field was applied to previously segmented GM images to warp non-linear transformation to standardized MNI space. During the transformations, the total amount of GM was preserved. All images were smoothed using an 8-mm full width half-maximum Gaussian kernel.

We first used *F*-contrast to test voxel-wise differences in GM volume among the three SA groups. For exploratory purposes, the *F*-test result was examined in a liberal cluster-defining threshold (*z* = 2.33, *k* > 500). The main effect of the *F-map* indicated differences in volumes of regional GM among the three SA groups. Based on the directional hypothesis that younger SA would be associated with larger brain volumes, we additionally conducted three pairwise *t*-tests (younger > same, same > older, younger > older). We identified the *post hoc* test results based on whether the voxels above cluster-level (cluster defining threshold of *z* = 3.09) or voxel-level family-wise error (FWE) *p* < 0.05 were included in the aforementioned clusters of *F*-test results. The cluster-level FWE rate was estimated based on Gaussian random field theory. Each VBM analysis was conducted after adjusting for age, gender, education and TIV.

### Age-Prediction Datasets

To estimate the degree of the age-related brain structural changes that have occurred in an individual, we implemented out-of-sample modeling and the prediction scheme that has recently been used to estimate brain ages (Franke et al., 2010; Liem et al., 2017; Rudolph et al., 2017). We utilized two publicly assessable datasets which consisted of T1-weighted MRI images: The Open Access Series of Imaging Studies (OASIS^1^) and The Information eXtraction from Images (IXI^2^). The cross-sectional (Marcus et al., 2007) and longitudinal datasets (Marcus et al., 2010) of the OASIS consisted of 512 healthy adults aged 18–94 (mean age = 51.64, SD = 24.91, female: 256). Subjects diagnosed with dementia (clinical dementia rating ≥ 0.5) were excluded from the OASIS dataset. The IXI dataset consisted of 545 healthy adults aged 19–86 (mean age = 48.78, SD = 16.53, female: 342). To reduce non-linear age effects in model construction (Fjell et al., 2013, 2014) and avoid biased modeling in predicting subjects of KSHAP who have a relatively old age range (59–84), we selected subjects above the age of 40 as the training sample. The finalized age-prediction data included 598 subjects (mean age = 63.28, SD = 12.97, female: 383).

### Age-Prediction Image Preprocessing

To apply a standardized preprocessing analysis pipeline across different MRI scan protocols in both KSHAP and the age-prediction datasets, we used a fully automated preprocessing procedure implemented in CAT12 r1113 (Computational Anatomy Toolbox, Structural Brain Mapping Group, Departments of Psychiatry and Neurology, Jena University Hospital^3^). First, a spatial-adaptive non-local means (SANLM) denoising filter (Manjón et al., 2010) was employed. Segmentation algorithms based on the adaptive maximum a posterior (AMAP) technique, implemented in CAT12, were used to classify brain tissue into three classes: GM, white matter (WM) and cerebrospinal fluid (CSF). Additionally, partial volume estimation (PVE) was used to create a more accurate segmentation for the two mixed classes: GM–WM and GM–CSF. Projection-based estimation of cortical thickness was conducted in the segmented images (Dahnke et al., 2012, 2013), which showed a comparable accuracy with other surface-based tools (Righart et al., 2017). In total, 156 values were extracted from CAT12 region of interest (ROI) analysis pipeline, including 148 cortical thickness and averaged GM density in eight bilateral subcortical structures (caudate, putamen, amygdala and hippocampus). Cortical areas were defined based on automatic parcellation of gyri and sulci (Destrieux et al., 2010), while subcortical volumes were defined using the Neuromorphometric atlas^4^. Identical procedures for preprocessing and extracting ROI values were made using the KSHAP data.

### Partial Least Square Regression Modeling

To effectively summarize and explain age-related characteristics of brain structure, we constructed a cross-validated partial least square regression (PLSR) model using the Caret package for R (Kuhn, 2015). PLSR reduces high-dimensional data into orthogonal components that have the greatest covariance with the output (the target of the prediction) before multiple regression analysis is conducted. In contrast to reducing dimensions with principal component analysis, PLSR decomposes orthogonal components in a way that is more relevant to the outcomes in the model construction stage. The PLS method is utilized in neuroimaging studies to effectively summarize the highly collinear data structures that are observed across brain regions (McIntosh and Lobaugh, 2004; Krishnan et al., 2011; Rudolph et al., 2017). In this study, the PLSR model was constructed to find linearly combined latent components highly predictive of the age of an individual. The training and estimation procedure of the age-prediction model was based on 156 ROI values from the open-access datasets (*n* = 598).

### Cross-Validation of the Age-Prediction Model

To construct a PLSR model applicable to the independent data and to achieve generalizability, we optimized the number of PLS components, using leave-one-out cross-validation (LOOCV). Although sequentially adding more components of latent variables would derive a more complex model in explaining the given data, cross-validation procedures must be applied to determine whether such a complex model ultimately explains the novel data that are independent from training data. Using the LOOCV procedure, we iteratively partitioned the training data (*n* = 597) to construct a model and predicted the left-out single-subject data. Each left-out procedure of modeling and predicting was repeated 598 times. Within each model, with differing number of components, the root mean squared error (RMSE) of the iterated LOOCV procedures was calculated. Examining the out-of-sample prediction error for each PLS component, the PLSR model was optimized between under-fitted and over-fitted models (Whelan and Garavan, 2014; Gabrieli et al., 2015; Rudolph et al., 2017). This approach identified the optimal model that showed the lowest RMSE and the greatest explained variance (*R*^2^) in predicting the age of the left-out subject.

### Statistical Analysis of Predicted Age

The brain ages of the individual subjects from the KSHAP data (*n* = 68) were predicted based on the weights of the cross-validated PLSR model, using the inputs for 156 brain regional values. We examined bivariate correlations between the real ages and predicted ages for the KSHAP data. Then, we used analysis of covariance (ANCOVA) to compare the differences in the predicted brain age between the SA groups, adjusting for the linear effects of gender, education and age. The adjusted mean of predicted age indicated the difference. We additionally examined whether the ANCOVA result changed when self-rated health, depressive symptoms, cognitive function and personality traits were additionally included as covariates. We determined the significance of the group difference at *p* < 0.05 level.

## Results

Rank-order correlation analysis showed that SA group was positively (from younger to older) associated with fewer years of education, lower working memory performance and poorer self-rated health (Table 1). Group comparisons specifically showed significant differences in chronological age (same < older; *p* = 0.043), depressive symptoms (same < older; *p* = 0.035) and MMSE-DS (younger, same > older; *p* = 0.013, *p* = 0.002, respectively).

**Table 1:** Demographic, psychosocial and cognitive test characteristics of the participants.

	Subjective age group			Total	Spearman’s correlation
	Younger (*n* = 29)	Same (*n* = 19)	Older (*n* = 20)		
Age	70.93 (6.32)	69.58 (5.96)	73.75 (6.54)	71.38 (6.41)	0.170
Gender	15:14	14:5	13:7	42:26	−0.136
Education	7.79 (4.47)	6.63 (3.11)	4.40 (3.05)	6.47 (3.95)	−0.323**
Depressive symptoms	11.45 (6.72)	10.42 (5.80)	14.90 (6.73)	12.18 (6.64)	0.198
Self-rated health	3.07 (0.96)	3.26 (1.19)	3.80 (1.06)	3.34 (1.09)	0.317**
Extraversion	34.59 (3.59)	34.68 (3.93)	33.45 (4.51)	34.28 (3.95)	−0.033
Openness	30.62 (3.73)	31.05 (3.29)	29.45 (3.80)	30.40 (3.64)	−0.097
MMSE	26.90 (2.30)	27.63 (2.34)	25.00 (3.06)	26.54 (2.73)	−0.232
Episodic memory	1.74 (0.55)	1.76 (0.39)	1.50 (0.41)	1.67 (0.48)	−0.193
Working memory	1.02 (0.34)	1.11 (0.33)	0.80 (0.26)	0.98 (0.33)	−0.248*
Category fluency	29.52 (9.43)	30.37 (8.32)	25.15 (7.5)	28.47 (8.75)	−0.145
Predicted brain age	73.24 (4.94)	75.03 (4.31)	77.15 (5.10)	74.89 (5.03)	0.413**

Higher self-rated health denotes poorer health. MMSE, Mini-mental State Examination. Spearman’s rho indicates rank-order correlation from younger to older subjective age. **p < 0.01, *p < 0.05.

**Figure 1:**
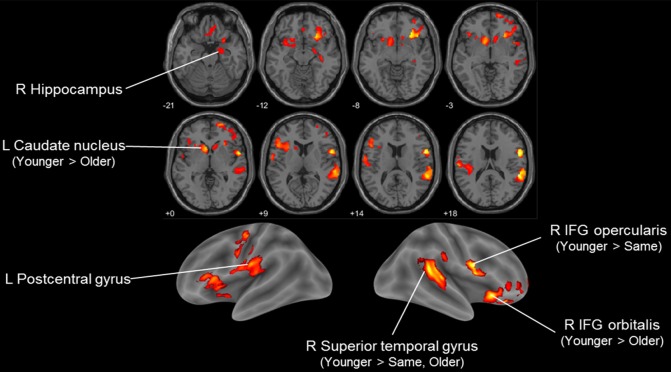
Voxel-based morphometry (VBM) *F*-test result comparing three subjective age (SA) groups (younger, same and older) in Korean Social Life, Health and Aging Project (KSHAP) data (*n* = 68). Significant group differences in regional gray matter (GM) density are visualized (*p* < 0.01, uncorrected, *k* > 500). *Post hoc* pairwise *t*-tests of the three groups indicated whether family-wise error (FWE)-corrected (voxel-level or cluster-level *p* < 0.05) voxels were included in the initially identified *F*-test clusters.

We then examined how SA was associated with regional GM volume using VBM analysis. Group differences among SA groups in the exploratory ANCOVA analysis showed differences in regional volume in the right inferior frontal gyrus, right superior temporal gyrus, bilateral striatum and left postcentral gyrus (Figure 1, Table 2). A *post hoc* comparison between each pair of groups identified four significant clusters among the *F*-test results (voxel or cluster-level FWE *p* < 0.05). Pairwise comparison showed that those with younger SA had especially larger regional GM density compared to those in the same or older SA group.

**Table 2:** Voxel-based morphometry (VBM) result comparing three subjective age (SA) groups in Korean Social Life, Health and Aging Project (KSHAP) data (*n* = 68).

	Cluster-level		Peak-level		MNI coordinates			*Post hoc*
Brain regions	*p* (FWE)	*k*	*p* (FWE)	*F*	*x*	*y*	*z*
R IFG (p. Opercularis)	0.424	1268	0.039	15.934	56	5	20	Younger > Same
R Superior temporal gyrus/Supramarginal gyrus	0.013	3455	0.050	15.486	56	−39	18	Younger > Same, Older
R IFG (p. Orbitalis)/Insula	0.016	3320	0.292	12.204	29	30	−8	Younger > Older
L Caudate nucleus	0.033	2855	0.624	10.474	−6	15	0	Younger > Older
L Postcentral gyrus	0.059	2487	0.654	10.339	−60	−18	21	
R Hippocampus	0.898	643	1.000	6.804	36	−21	−12	

*Regional gray matter (GM) density with significant group differences (*p* < 0.01, uncorrected, *k* > 500). *Post hoc* pairwise *t*-test indicated whether family-wise error (FWE)-corrected (voxel-level or cluster-level *p* < 0.05) voxels were included in the initially identified *F*-test clusters*.

**Figure 2:**
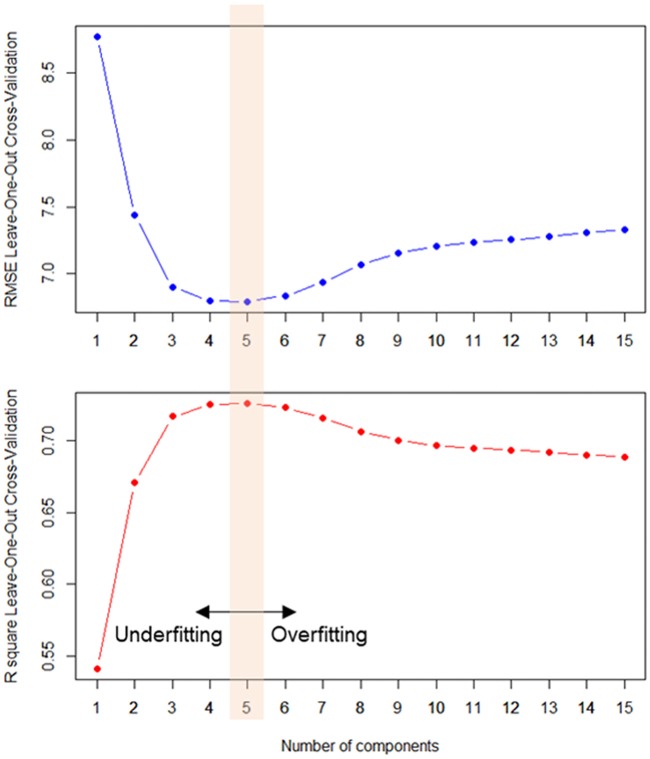
Averaged prediction error (root mean squared error, RMSE, upper panel) and variance explained (*R*^2^, lower panel) in every left-out training sample (*n* = 598) across the number components. Partial least square regression (PLSR) model with five latent constructs showed the most accurate out-of-sample age prediction.

**Figure 3:**
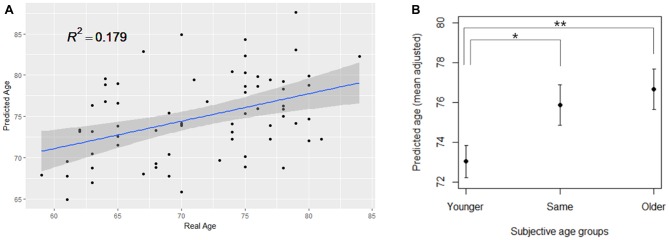
**(A)** PLSR model significantly predicting the real age of the KSHAP dataset (*n* = 68). **(B)** SA group differences in the predicted brain age. The group means of predicted brain age are adjusted for gender, education and real age in analysis of covariance (ANCOVA). Asterisks denote significant differences between groups. Error bars denote standard errors of the mean. **p < 0.01, *p < 0.05.

From the open-access database (*n* = 598), we constructed a PLSR model of brain structural morphology predicting chronological age. Models with sequentially added latent variables (from 1 to 15 components) were cross-validated using LOOCV. The model with five components had the greatest accuracy in predicting left-out data (Figure 2; RMSE = 6.795, *R*^2^ = 0.726). The PLSR models with more or less than five components showed relatively larger RMSE and smaller explained variance in predicting the left-out data, which indicated that these were either under-fitted or over-fitted models. The bilateral hippocampus, superior temporal gyrus, and inferior prefrontal cortex had the highest average of all coefficient weights, indicating the brain structures important in predicting the chronological age of the individuals in the final cross-validated model (Table 3). From the cross-validated PLSR model, the chronological age of the KSHAP subjects (*n* = 68) were predicted with a moderate accuracy (Figure 3A, *R*^2^ = 0.179, *p* = 3.32 × 10^4^, Mean absolute error = 5.74). Subjects in their 60 s had relatively higher predicted ages, whereas those in their 80 s showed estimated ages lower than their real ages.

**Table 3:** Average coefficients of the weights in the age-prediction model.

Rank	Brain regions	Average coefficients of PSLR components	Correlation coefficient (*r*) with age
1	L hippocampus	−3.718	−0.635
2	R hippocampus	−3.469	−0.613
3	L superior temporal gyrus	−2.930	−0.501
4	R superior temporal gyrus	−2.810	−0.595
5	R transverse temporal sulcus	−2.152	−0.408
6	L inferior frontal gyrus opercularis	−2.115	−0.616
7	R superior temporal gyrus	−1.990	−0.433
8	R inferior frontal gyrus triangularis	−1.953	−0.532
9	L superior temporal gyrus	−1.899	−0.552
10	L inferior insula	−1.872	−0.447
11	R inferior frontal opercular part	−1.801	−0.535
12	R parahippocampal gyrus	−1.796	−0.442
13	R inferior insula	−1.667	−0.432
14	R superior temporal gyrus	−1.662	−0.553
15	R inferior frontal triangular sulcus	−1.619	−0.340
16	L parahippocampal gyrus	−1.612	−0.407
17	L inferior frontal orbital part	−1.609	−0.395
18	R middle frontal gyrus	−1.582	−0.542
19	R superior temporal gyrus	−1.431	−0.539
20	R inferior frontal orbital part	−1.408	−0.329

*Among 156 regions of interest (ROIs) of regional cortical thickness and subcortical volumes, 20 ROIs with the highest weights are noted in descending order*.

The ANCOVA result showed a significant difference between SA groups in the predicted age, when gender, education and chronological age were adjusted for (*F*_(2,62)_ = 4.441, *p* = 0.016, *η*^2^ = 0.125). Spearman’s correlation analysis showed that ordinal ranking from younger to older was positively associated with predicted brain age when the effect of chronological age was partialled out (*ρ* = 0.314, *p* = 0.009). As shown in Figure 3B, a *post hoc* test showed significant differences in predicted age between the younger SA group and the other two groups (younger > same, *p* = 0.039; younger > older, *p* = 0.009) but the difference between the same and older SA was not significant (same > older, *p* = 0.558). We repeated the ANCOVA tests by adding covariate terms for depressive symptoms, cognitive functions, personality traits and self-rated health, all of which had been found to be associated with SA in previous studies. The ANCOVA results, however, remained unchanged, even when each covariate was entered (*p*s < 0.05, *η*^2^ = 0.105–0.142).

## Discussion

SA was associated with decreased regional GM volume and predicted brain age as well. Our findings suggest that feeling subjectively older than one’s age may reflect relatively faster aging brain structures, whereas those who feel subjectively younger would have better-preserved and healthier structures. This study, to our knowledge, was the first attempt to examine the neuroanatomical underpinnings of SA.

When we examined regional structural differences in GM using VBM analysis, we found that the volumes of the inferior prefrontal cortex, posterior superior temporal gyrus and striatal region showed the strongest association with SA groups. Previous studies have indicated that the volume of the right insula is associated with metacognition and awareness of task performance (Cosentino et al., 2015), and the right posterior temporal gyrus plays an important role in processing the awareness of one’s body and spatial representations (Karnath et al., 2001; Blanke et al., 2002). Neural degradation in these regions may affect how one tracks one’s physical state and one’s perception of age-related changes. On the other hand, the core mechanism of SA has been located in the fronto-striatal dopaminergic system, which plays a central role in explaining healthy brain aging and cognitive decline (Bäckman et al., 2010). Meanwhile reduced brain volumes of the inferior frontal cortex have been found to be associated with inefficient functioning of inhibitory control processing (Turner and Spreng, 2012; Aron et al., 2014). The ability to inhibit or suppress irrelevant or no-longer-relevant information has been proposed as a core process in explaining the age-related cognitive decline in a variety of tasks (Hasher and Zacks, 1988; Hasher et al., 1991). The deficiency in tasks requiring the function of cognitive control may have affected overall appraisal of one’s state of cognitive aging. It is notable, however, that other brain regions highly susceptible to aging (Fjell et al., 2009b, 2014) did not show a prominent association with SA in our results. That is, among the many brain regions undergoing age-related structural changes, specific areas were more relevant to explaining how older adults feel their own process of aging. It should also be noted that interpretations based on the variation of regional morphology and reverse inferences should be made with great caution (Poldrack, 2006).

To clarify the age-relatedness of the association between the brain structures and SA, we applied the out-of-sample modeling procedure to derive an overall index of brain aging. Since individual differences in brain structures can occur not only due to age-related neurophysiological changes but also from other pre-existing individual differences such as personality traits (Kanai and Rees, 2011), the application of the age-prediction modeling was crucial in interpreting the neuroanatomical differences observed in the study. Importantly, we found that age-related multivariate patterns expressed in cortical thickness and subcortical volumes differed among the SA groups. More specifically, the brain regions that were highly predictive of chronological age were found to be partly overlapping with the regions identified in the VBM analysis, including the hippocampus, superior temporal gyrus and inferior frontal cortex. Those who felt younger than their real age also had younger brain structural patterns and vice versa.

Our results may suggest that, in concert with the interoceptive hypothesis (Diehl and Wahl, 2010), feeling younger or older than one’s chronological age can be an indirect perception of neurobiological aging rather than a psychological defense against negative age stereotypes (Weiss and Lang, 2012) or social comparison (Mussweiler et al., 2000). If individual differences in SA result mainly from social impacts on attitudinal representation, it is less likely that feeling younger is associated with markers of neurobiological aging. Examining the correlates of SA using objectively measured aging markers other than self-reported measures may strengthen the validity of the interoceptive hypothesis. Although previous studies have already shown that those with older SA have poorer biological aging markers (Stephan et al., 2015b,c), our findings extend the hypothesis that older SA is associated with greater progression of brain aging process and poorer brain health. Significant tissue atrophy in the GM and older brain age may be reflective of cerebrovascular risks (Seo et al., 2012; Lockhart and DeCarli, 2014), and such changes may cause older adults to appraise their deteriorated functions as being a result of their aging (Vestergren and Nilsson, 2011).

Consistent with previous studies that have reported the clinical significance of subjective perceptions of cognitive decline (Rabin et al., 2017), subjective appraisal of one’s own decline may provide information on neurobiological changes not otherwise detectable with objective cognitive tests. If feeling older in one’s SA is affected by decreased cognitive function or cognitive efficacy (Boehmer, 2007; Schafer and Shippee, 2010; Stephan et al., 2011), it is likely that the SA reflects of pathological brain changes and subtle decreases in the neural capacity that cannot otherwise be detected. Diminished volumes of GM and older brain age may lead to reduced processing efficiency in a variety of demanding cognitive tasks, and this prolonged mismatch between reduced neural resources and burdensome environmental demands can create a subjective perception of aging. Individuals with older SA feel older because they experience frequent negative sensations as they make more cognitive efforts in daily life compared to those who report younger or same SA. Benign cognitive failures that occur daily tend to be attributed to age-related changes among older adults (Vestergren and Nilsson, 2011). It is possible that the effect of brain aging may influence the awareness of age-related change more directly than a mere appraisal of physical health (Cole and Franke, 2017), as seen from our result, which remained unchanged even when the effect of self-rated health was accounted for. Even though we observed that higher SA was associated with lower cognitive performance, especially in working memory function, adjustment of these differences did not change the relationship between the SA and brain age. While we assumed that SA reflects a stable and accurate perception of age-related changes in the brain, another possibility considered in this study was that people can feel older due to excessively self-referential and negative emotional states (Reid and MacLullich, 2006; Rabin et al., 2017). That is, when the experience of benign age-related cognitive decline is overestimated, an older adult can perceive him- or herself as older than their real age (Pearman et al., 2014; Hülür et al., 2015). However, according to the results of our study, neither self-rated health nor depressive symptoms accounted for the significant difference in brain age. These additional considerations may suggest that individual differences in SA stably reflect a prolonged and accumulated status of the brain aging to a certain degree that does not fluctuate in temporary conditions (Hughes et al., 2013; Stephan et al., 2013; Geraci et al., 2018).

Another notable finding was that the younger SA group showed a significant difference in predicted brain age. Although most previous studies have examined linear and continuous effects of SA on various outcomes, our findings suggest that feeling younger and feeling older may not be symmetric or linear cognitive processes (Kotter-Grühn and Hess, 2012; Weiss and Lang, 2012), and health consequences may also differ between the two categorized groups. In our study, while older SA group showed a tendency to have poorer cognitive function and exhibit greater depressive symptoms, feeling younger was especially associated with younger structural characteristics of the brain.

Several limitations should be noted. Although we constructed an age-predicting model that accurately explains real age across the left-out subjects at a comparable level with a previous study (Franke et al., 2010), the age-prediction model showed relatively lower accuracy among the KSHAP subjects. The low correlation between real age and estimated brain age may be explained by the fact that the predictive performance of external validation is typically poorer than that of internal validation because independent datasets do not guarantee homogeneous sample characteristics, data collection protocols and modeling parameters (Woo et al., 2017). Extensive screening procedures that are based on the neuropsychological assessments could have resulted in over-representing healthy older adults free from severe neuropathology in our study than when they were in the open access datasets. This in turn would have lowered the slope between real age and estimated brain age. Moreover, cross-sectional age effects can be underestimated especially when age ranges are confined within the 60 s and the 80 s (Fjell et al., 2014). If the KSHAP data had included midlife subjects, predicted ages may have been more accurate across subjects. Another limitation is the coarse measurement of SA. The low resolution in the current categorical measure may have resulted in the loss of information regarding the extent to which the participants feel about their age within each categorized SA groups. In addition, recent studies have underscored the multidimensional aspects of SA (Diehl et al., 2014), and attempted to additionally separate the concept of SA into negative stereotypes of aging (Levy et al., 2016) and self-identification based on the social reference group (Barak, 2009), other than interoceptive awareness. The interpretation could have been clearer if we had questioned both the aspects of social influence and of the internal awareness of SA separately. Further investigation is required to distinguish specific neural mechanisms of both interoceptive perception and social influence. Lastly, although we have mainly interpreted the SA as being a result of age-related brain change, maintaining younger SA may also lead to a lifestyle physically and mentally more active, which leads to healthier brain. Future longitudinal studies will further elucidate these temporal relationships.

## Author Contributions

JC and YY obtained funding and supervised the study. SK: data collection, study design, data analysis and manuscript writing. HK: data collection and data analysis.

## Conflict of Interest Statement

The authors declare that the research was conducted in the absence of any commercial or financial relationships that could be construed as a potential conflict of interest.

## Footnotes

^1^http://www.oasis-brains.org

^2^http://brain-development.org/ixi-dataset

^3^http://dbm.neuro.uni-jena.de/cat/

^4^http://Neuromorphometrics.com
